# Methods for assessing change in brain plasticity at night and psychological resilience during daytime between repeated long-duration space missions

**DOI:** 10.1038/s41598-023-36389-6

**Published:** 2023-07-05

**Authors:** Kuniaki Otsuka, Germaine Cornelissen, Yutaka Kubo, Koichi Shibata, Koh Mizuno, Tatsuya Aiba, Satoshi Furukawa, Hiroshi Ohshima, Chiaki Mukai

**Affiliations:** 1 Space Biomedical Research Group, Japan Aerospace Exploration Agency, Ibaraki, Japan; 2 Halberg Chronobiology Center, University of Minnesota, Minneapolis, MN USA; 3 Tokyo Women’s Medical University, Tokyo, Japan; 4 Faculty of Education, Tohoku Fukushi University, Miyagi, Japan; 5 Tokyo University of Science, Tokyo, Japan

**Keywords:** Neuroscience, Physiology, Psychology, Environmental sciences, Planetary science, Cardiology, Health care, Astronomy and planetary science, Engineering

## Abstract

This study was designed to examine the feasibility of analyzing heart rate variability (HRV) data from repeat-flier astronauts at matching days on two separate missions to assess any effect of repeated missions on brain plasticity and psychological resilience, as conjectured by Demertzi. As an example, on the second mission of a healthy astronaut studied about 20 days after launch, sleep duration lengthened, sleep quality improved, and spectral power (ms^2^) co-varying with activity of the salience network (SN) increased at night. HF-component (0.15–0.50 Hz) increased by 61.55%, and HF-band (0.30–0.40 Hz) by 92.60%. Spectral power of HRV indices during daytime, which correlate negatively with psychological resilience, decreased, HF-component by 22.18% and HF-band by 37.26%. LF-component and LF-band, reflecting activity of the default mode network, did not change significantly. During the second mission, 24-h acrophases of HRV endpoints did not change but the 12-h acrophase of TF-HRV did (P < 0.0001), perhaps consolidating the circadian system to help adapt to space by taking advantage of brain plasticity at night and psychological resilience during daytime. While this N-of-1 study prevents drawing definitive conclusions, the methodology used herein to monitor markers of brain plasticity could pave the way for further studies that could add to the present results.

## Introduction

Herein, we illustrate our method for investigating brain plasticity of an astronaut by assessing how sleep performance and heart rate variability (HRV), gauging activity of intrinsic networks of the brain, particularly the default mode network (DMN) and salience network (SN), changed from first-time to second-time long-duration spaceflight 4 years later.

Magnetic resonance imaging (MRI) studies showed narrowing of the central sulcus, upward shift of the brain, and narrowing of cerebrospinal fluid spaces at the vertex in most astronauts examined^1–9^. Impaired cerebrovascular circulation in microgravity may induce cortical reorganization. Understanding the effects of spaceflight on the human central nervous system is pivotal for the development of adequate countermeasures. Maximizing crew performance and health is crucial for the success and safety of future prolonged space missions, including missions to the moon or Mars^10–12^.

The central nervous system seems capable of adaptation to microgravity by the process of neuroplasticity, as previously shown in animals^13^. Yet, little is known about the effects of microgravity and gravity transitions on the human brain^14^. After exposure to microgravity, significant differences in resting-state functional connectivity between motor cortex and cerebellum, and changes within the DMN have been reported^2,14^. Changes in brain function could account for the fact that second-time flyers are less prone to some microgravity-related problems than first-time flyers, given the process of neural adaptation, as conjectured by some^14–16^. It is thus important to learn how long physiological adaptation processes last. Research investigating space travelers at different intervals post-flight could answer this question.

The intimate brain–heart connection enunciated by Claude Bernard can be studied by analyzing HRV^17^. HRV may reflect the activity of the coordinating system^18–21^, notably brain functional connectivity, including the DMN and the SN, which integrates the brainstem nuclei that directly regulate the heart. The heart and brain are connected bi-directionally, and HRV varies in concert with changes in brain functional connectivity. As we reported earlier^19,20^, HRV may serve as a proxy for ‘vertical integration’ of the brain in association with DMN and SN functions. HRV may provide information on how the brain coordinates with the periphery, and may thus inform about the extent of adaptive adjustment and brain plasticity^17^. Accordingly, herein, we analyze HRV in the course of space missions to gain knowledge about brain plasticity and adaptation to space^19–22^.

Many investigations recently showed how psychological resilience can be assessed using imaging modalities within the brain, such as low-frequency fluctuations in the anterior cingulate cortex (ACC), orbitofrontal cortex (OFC), posterior cingulate cortex (PCC) and thalamus, largely comprising the brain functional networks of the DMN and SN^23,24^. Astronauts aboard the International Space Station (ISS) are well motivated and become even more so with subsequent missions.

## Subject and methods

### Subject

A healthy astronaut participated in the ISS Japan Aerospace Exploration Agency (JAXA) investigation named “Biological Rhythms 24 Hrs & 48 Hrs”. Stays in space lasted approximately 4.5 and 6 months on the first and second flights, respectively. The astronaut had passed class III physical examinations from the National Aeronautics and Space Administration (NASA). The study was approved by the Institutional Review Boards of NASA, ESA (European Space Agency), Pro0406 (MODCR940)—Amd-10, and JAXA, JX-IRBA-20-084 Amd-10. Informed consent was obtained from the astronaut. A detailed explanation of the study protocol was given to the astronaut before obtaining written, informed consent, according to the Declaration of Helsinki Principles. All methods were performed in accordance with the JAXA/ESA/NASA guidelines and regulations.

### Experimental protocols

Ambulatory around-the-clock ECG records were obtained over 24 h on the first flight and over 48 h on the second flight by a two-channel Holter recorder (FM-180; Fukuda Denshi, Tokyo, Japan). Measurements were taken four times during each mission: once before flight, twice during flight on the ISS (ISS01 and ISS02), and once after the mission (Post) (Mission 1/2: Pre: 189/50 days before launch; ISS01 and ISS02: day 18/21 and day 67/181 after launch, respectively; Post: 138/188 days after return to Earth).

For assessing microgravity-induced brain plasticity, we focused only on ISS01 because measurements were obtained at about the same time after launch in both missions, on days 18 and 21 on the ISS, respectively. Because the second session on the ISS took place much later on the second than on the first spaceflight (on day 181 versus day 67), data collected during ISS02 were not suitable for analysis.

### Analysis of HRV

Data collection and measurement procedures were conducted as previously reported^19,20,25–28^. Briefly, for HRV measurements, the RR intervals between normal QRS waveforms were extracted as normal-to-normal (NN) intervals, which were A/D converted (125-Hz) with 8-ms time resolution. The authors first confirmed that all artifacts were actually removed and that the data excluded supraventricular or ventricular arrhythmia. First, time-domain measures (CVRR, r-MSSD and pNN50), Lorenz plot (Length, Width and Length/Width ratio), and conventional frequency-domain measures (TF-, ULF-, VLF-, LF- and HF-HRV and LF/HF ratio)^29^ and β, reflecting the intrinsic cardiovascular regulatory system, were obtained with the Maximum Entropy Method (MEM) software (MemCalc/CHIRAM, Suwa Trust GMS, Tokyo, Japan)^30^. Time series of NN intervals covering 5-min intervals were analyzed by the MEM to compute the spectral power in different frequency regions. Next, HRV measures reflecting dynamics of brain functional connectivity^17–19,23,24,31–36^ were assessed, as defined in the lower part of Table 1. Frequency regions examined were 0.05–0.15 Hz (LF-component) and 0.15–0.50 Hz (HF-component), according to Chang et al.^18^; 0.01–0.05 Hz (LF-band), 0.05–0.10 Hz (MF1-band), 0.10–0.15 Hz (MF2-band), and 0.15–0.20 Hz (HF01-band), according to Baria et al.^37^; 0.20–0.30 Hz (HF02-band), 0.30–0.40 Hz (HF03-band), and 0.40–0.50 Hz (HF04-band), according to Chen and Glover^38^.

**Table 1:** Frequency-domain measures of heart rate variability (HRV).

Frequency-domain measures (units, ms^2^)		Frequency range (Hz)	Description	Related references	Brief physiologic correlation
Conventional frequency-domain measures of HRV	TF-HRV	0.0001–0.50	Variance of all N–N intervals over 3-h interval	^29^	Index suggestive of anti-aging or longevity
	ULF-HRV	0.0001–0.003	Power in ULF range. Fluctuations in N–N intervals with underlying cycle length [> 5 min − ≤ 3 h]		Predominantly 3-h rhythm, but other influences, including activity and neuroendocrine rhythms, contribute to ULF. Circadian rhythms, core body temperature, metabolism, hormones, and intrinsic rhythms generated by the heart all contribute to ULF
	VLF-HRV	0.003–0.04	Power in VLF range, Infra-slow oscillation (ISO)		VLF is the most predictive of adverse outcomes, including all-cause motality. Historically, VLF may reflect both vagal control of heart rate and also the effect of the renin-angiotensin system. Recently, VLF rhythm appears to be produced by the heart itself and may be an intrinsic rhythm that is fundamental to health and well-being
	LF-HRV	0.04–0.15	Power in LF range		LF primarily reflects baroreflex activity while at rest
	HF-HRV	0.15–0.40	Power in HF range		Relative vagal modulation of heart rate in response to respiration. Higher values reflect higher parasympathetic (vagal) influence or greater degree of erractic rhythm
HRV measures reflecting dynamics of brain functional connectivity	LF-component	0.05–0.15	Power in LF-component range	^18,19^	Index reflecting the Default Mode Network (DMN) circuit, acting via the Temporo-Parietal Junction networks. Increase in LF-component suggests DMN’s role in adaptation to novel environment and in coordinating functional connections with other brain networks, including the Salience Network
	HF-component	0.15–0.50	Power in HF-component range		Index reflecting the Salience Network circuit, acting via the lateral orbitofrontal cortex loop, involved in the adaptation process. Increases in HF-component are accompanied by increases in functional connectivity between the dorsal anterior cingulate cortex and regions including the basal ganglia, thalamus, midbrain and brainstem, and between the amygdala and regions including the basal ganglia, anterior insula and dorsolateral prefrontal cortex
	LF-band	0.01–0.05	Power in LF-band, Infra-slow oscillation (ISO)	^19,37^	Index primarily related to brain’s DMN activity, in medial prefrontal and precuneus/posterior cingulate cortex parts
	MF1-band	0.05–0.10	Power in MF1-band range, ISO		Index primarily related to brain’s DMN activity, mainly in the thalamus and basal ganglia
	MF2-band	0.10–0.15	Power in MF2-band range		Index primarily related to brain’s DMN activity, mainly in the orbitofrontal, insular and temporal cortex parts
	HF01-band	0.15–0.20	Power in HF1-band range		Index primarily related to medial orbitofrontal cortex (mOFC)/medial prefrontal cortex (mPFC)-guided core integration system
	HF02-band	0.20–0.30	Power in HF2-band range	^17,19,31–34,38^	Index reflecting relative vagal modulation of heart rate in response to respiration
	HF03-band	0.30–0.40	Power in HF3-band range		Index reflecting psychological resilience related to brain’s Salience Network activity, primarily in anterior cingulate cortex on subjective well-being
	HF04-band	0.40–0.50	Power in HF4-band range		Index reflecting psychological resilience related to brain’s Salience Network activity, primarily in anterior cingulate cortex on subjective well-being
	Infraslow oscillation (ISO) at night	0.01–0.10	Power in frequency range of 0.01–0.10 Hz	^20,23,24,35,36^	HRV index coordinating brain dynamics via thalamic astrocytes plays key role in adaptation to novel environments; 0.02 Hz or 1/min fluctuations underly unconscious processing of information among resting state networks
	LFFs (low-frequency fluctuations) during daytime				Recently, LFFs are noted HRV indices reflecting psychological resilience related to brain’s DMN activity, primarily in orbitofrontal cortex on subjective well-being

A positive response in these bands is thought to indicate how astronauts adapt to the space environment. Increases in the LF- and MF1-bands reflect an activation of the DMN’s medial prefrontal cortex (mPFC), posterior parietal cortex, posterior portion of precuneus and posterior cingulate cortex. Changes in the MF2- and HF01-, HF02-, HF03-, and HF04-bands show dynamic interactions among the DMN and SN, i.e., the alerted DMN involved in the adaptation to a novel environment^18,19,37,38^.

### Cosine curve fitting for estimating amplitude and phase by cosinor

The MEM software (MemCalc/Win, Suwa Trust GMS, Tokyo, Japan)^30^ was used to fit a single 24-h or 12-h cosine curve individually to each of the HRV measures by cosinor^39–41^. The 24-h and 12-h amplitudes and acrophases together with the MESOR (Midline Estimating Statistic Of Rhythm, a rhythm-adjusted mean) were thereby estimated. Changes in biological rhythm amplitude and acrophase assessed the response in rhythmicity of each biological rhythmic component to the space environment.

### Sleep duration and sleep quality

**Figure 1:**
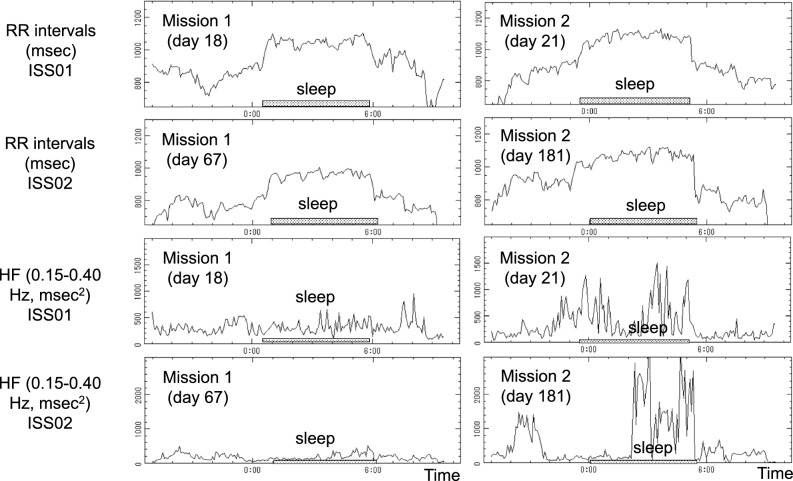
Estimation of sleep span and assessment of sleep quality. RR-intervals (first two rows) and HF-HRV (last two rows) of the first (left) and second (right) spaceflight assess sleep duration and sleep quality during ISS01 and ISS02, respectively. HF-HRV spectral power, reflecting sleep quality, is clearly larger on the second than on the first spaceflight during both ISS01 and ISS02. Sleep-related increase in RR-intervals also appears to be larger on the second than on the first spaceflight.

Sleep duration at night was estimated by using circadian profiles of RR-intervals and 5-min HF endpoints of HRV^25,28,29,42^. Sleep duration on the second spaceflight was estimated as the average of the two consecutive sleep spans of the 48-h ECG record. Sleep quality was determined based on whether a sleep-related increase in RR-interval and in HF of HRV could be observed or not, as shown in Fig. 1. For reference, results during ISS02 are also depicted.

### Statistical analyses

Data shown in Table 2 are expressed as mean ± standard error (SE). The ECG recording was started at 13:25 during the 1st space-flight mission, and at 17:50 during the 2nd space-flight mission. For comparison of HRV indices, statistical analyses were applied on hourly averages of the 5-min estimates in order to minimize serial correlation. Paired hourly HRV indices were compared between the two spaceflights, focusing on ISS01 (days 18 and 21 after launch, respectively), using the paired t-test. Cohen’s distance was determined to assess effect size. The Stat Flex (Ver. 6) software (Artec Co., Ltd., Osaka, Japan) was used. P-values less than 0.05 were considered to indicate statistical significance.

**Table 2:** Comparison of heart rate variability between the two spaceflights suggests role of brain functional network for faster adaptation to microgravity on second spaceflight.

	Unit, or frequency range (Hz)	Day time (awake)							Night time (asleep)						
		n	Mission 1 (day 18 after launch)		Mission 2 (day 21 after launch)		Paired t-test		n	Mission 1 (day 18 after launch)		Mission 2 (day 21 after launch)		Paired t-test	
			Mean	SE	Mean	SE	t-value	p-value		Mean	SE	Mean	SE	t-value	p-value
HR	bpm	18	75.9	2.7	71.4	1.7	− 1.415	0.1752	6	58.3	1.0	56.5	1.1	− 3.277	0.0220
NN	ms	18	813.3	21.8	849.9	17.5	1.330	0.2012	6	1032.1	16.5	1065.3	19.7	3.100	0.0269
β	0.0001–0.01	17	− 1.2065	0.0875	− 0.9793	0.0465	1.799	0.0953	6	− 0.7533	0.0732	− 0.5400	0.0991	3.719	0.0137
CVRR	ms	18	8.08	0.30	7.40	0.44	− 1.178	0.2549	6	5.67	0.30	5.79	0.28	0.521	0.6246
r-MSSD	ms	18	41.8	2.2	32.2	1.9	− 4.697	**0.0002**	6	45.7	1.2	52.0	5.2	1.191	0.2869
pNN50	%	18	17.7	1.4	9.2	1.4	− 5.178	**0.0001**	6	20.4	1.0	15.4	3.4	− 1.481	0.1986
Length	ms	18	300.2	12.1	263.6	8.9	− 2.252	0.0378	6	302.1	18.6	305.2	11.9	0.151	0.8857
Width	ms	18	138.2	18.8	81.9	5.7	− 2.965	0.0087	6	119.3	3.5	159.3	17.6	2.629	0.0466
Len/Wid	–	18	2.62	0.09	3.69	0.25	4.416	**0.0004**	6	2.55	0.09	2.42	0.31	− 0.390	0.7129
TF	0.0001–0.50	17	8857.4	1400.4	5351.3	693.0	− 1.871	0.0840	6	4238.2	460.1	4498.2	348.7	0.656	0.5408
ULF	0.0001–0.003	17	6227.8	1479.7	2672.4	583.3	− 1.846	0.0878	6	1168.7	223.1	879.4	160.7	− 1.961	0.1071
VLF	0.003–0.04	18	1566.0	112.6	1642.1	129.6	0.482	0.6360	6	1724.7	172.8	2083.1	284.0	1.327	0.2419
LF	0.04–0.15	18	920.2	61.1	872.4	48.5	− 0.703	0.4919	6	999.7	142.6	978.2	108.8	− 0.210	0.8422
HF	0.15–0.40	18	283.1	29.9	227.7	26.4	− 2.108	0.0501	6	317.4	20.5	510.7	88.9	2.064	0.0939
LF/HF	–	18	3.63	0.20	4.85	0.29	4.266	**0.0005**	6	3.44	0.51	2.62	0.30	− 1.236	0.2714
LF-component	0.05–0.15	18	783.2	50.9	747.0	42.3	− 0.617	0.5451	6	819.3	113.1	809.2	104.1	− 0.151	0.8859
HF-component	0.15–0.50	18	321.0	34.1	249.8	29.1	− 2.479	0.0240	6	345.1	20.3	557.5	97.1	2.095	0.0903
LF-band	0.01–0.05	18	1035.6	86.7	1052.0	60.6	0.198	0.8453	6	1448.7	152.3	1531.0	187.8	0.402	0.7040
MF1-band	0.05–0.10	18	522.1	34.1	462.0	23.0	− 1.623	0.1230	6	643.9	101.3	635.4	86.3	− 0.183	0.8619
MF2-band	0.10–0.15	18	261.1	18.7	285.1	21.6	0.938	0.3612	6	175.4	15.5	173.8	26.7	− 0.060	0.9548
HF01-band	0.15–0.20	18	111.1	10.9	95.4	9.0	− 1.418	0.1743	6	102.5	10.2	138.3	17.5	2.256	0.0737
HF02-band	0.20–0.30	18	113.5	13.1	95.6	14.5	− 1.415	0.1750	6	162.2	13.5	270.9	55.1	1.757	0.1392
HF03-band	0.30–0.40	18	58.5	6.9	36.7	5.2	− 4.791	**0.0002**	6	52.7	2.1	101.5	20.8	2.338	0.0665
HF04-band	0.40–0.50	18	37.9	4.5	22.1	3.1	− 5.531	**0.0003**	6	27.7	0.9	46.8	9.0	2.203	0.0788

Tests applied on hourly averages of 5-min intervals in order to eliminate or at least reduce serial correlation.

*n* number of hourly averages (of 5-min intervals), *SE* standard error.

P-values not adjusted for multiple testing; P < 0.05 after adjusting for mutiple testing highlighted in bold.

## Results

### Sleep performance

While sleep duration around day 20 after launch during ISS01 cannot be statistically compared between the two missions without knowing its day-to-day variation for this astronaut, it was more than one hour longer on the second than on the first mission (374 vs. 300 min during ISS01 and 365 vs. 295 min during ISS02). Such large differences between the two missions are not seen before launch (297 vs. 289 min) and after return to Earth (330 vs. 360 min). Sleep quality may also have been improved on the second compared to the first spaceflight, as suggested by a clear increase in spectral power of the HF-HRV, Fig. 1.

### Dynamic response of the autonomic nervous system

As shown in Table 2 (right), nighttime HR was lower by about 2 bpm on the second than on the first mission during ISS01 (56.5 vs. 58.3 bpm). A larger change occurred in the intrinsic cardiovascular regulatory function |β| (0.5400 vs. 0.7533). Parasympathetic activity was increased on the second mission, as shown by the Width of Lorenz plot (159.3 vs. 119.3) and by HF-HRV (510.7 vs. 317.4 msec^2^). Such changes, well exceeding 25% are of sufficient magnitude to serve as biomarkers in future studies.

During daytime (Table 2, left), parasympathetic activity was lower on the second than on the first mission during ISS01, gauged by r-MSSD (32.2 vs. 41.8), pNN50 (9.2 vs. 17.7), Length of Lorenz plot (263.6 vs. 300.2), Width of Lorenz plot (81.9 vs. 138.2), and HF-HRV (227.7 vs. 283.1). Sympathetic activity increased during daytime, gauged by the LF/HF ratio (4.85 vs. 3.63) and Lorenz plot’s Length/Width (3.69 vs. 2.62). Again, these changes are non-negligible, extending from over 10% to about 50%.

### Brain functional networks estimated by heart rate variability during spaceflight

**Figure 2:**
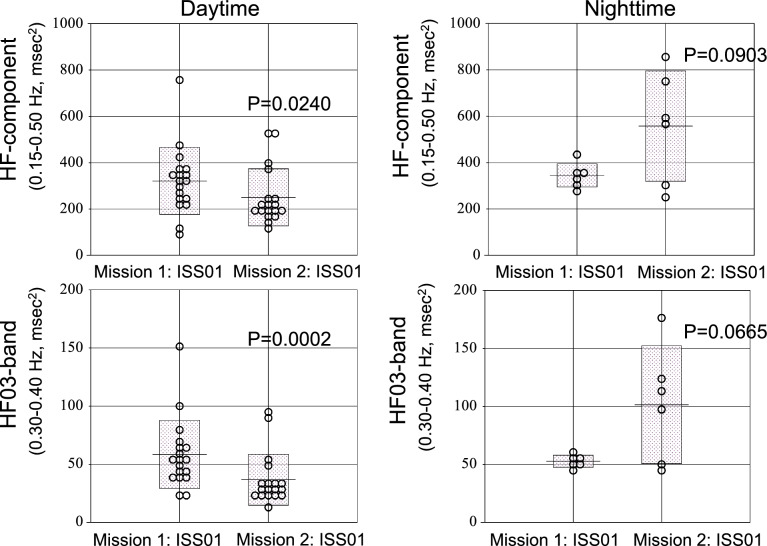
Brain plasticity at night and psychological resilience during daytime took place on the second mission. HRV indices reflecting SN activity, HF-component and HF03-band, decreased during daytime (left), but increased during nighttime (right). These changes are in agreement with previous investigations showing that brain plasticity takes place at night while psychological resilience takes place during the daytime (see text).

Activity of brain functional networks is reflected in several indices of HRV^17–19,31–35^. Although not statistically significant, HRV indices reflecting SN activity tended to increase on the second compared to the first spaceflight during nighttime (Table 2, right)^17,32,37,43^. The HF-component increased by 62% (from 345.1 to 557.5 ms^2^) (Fig. 2, top right), HF01-band by 35% (from 102.5 to 138.3 ms^2^), HF02-band by 67% (from 162.2 to 270.9 ms^2^), HF03-band by 92% (from 52.7 to 101.5 ms^2^) (Fig. 2, bottom right), and HF04-band by 69% (from 27.7 to 46.8 ms^2^). These HRV indices changed statistically significant during daytime, (Table 2, left). The HF-component decreased by 22% (Fig. 2, top left), HF03-band and HF04-band decreased by 37% (from 58.5 to 36.7) (Fig. 2, bottom left) and by 42% (from 37.9 to 22.1), respectively. Corresponding effect sizes are medium or large based on Cohen’s distance. It should be possible to detect changes of this magnitude in future studies.

### Assessment of circadian and circasemidian components of HRV endpoints

Circadian and circasemidian amplitudes of HRV endpoints are shown in Table 3. On the first spaceflight, the circadian amplitude of HR increased more than two-fold both during ISS01 (255%) and ISS02 (271%) compared to pre-flight, as observed previously^28^. This was not the case on the second spaceflight. On the first mission, the circadian amplitude of the intrinsic cardiovascular regulatory system function (β) also increased during both ISS01 (303%) and ISS02 (233%), compared to pre-flight. The adaptation behavior of the 12-h component of HRV endpoints was remarkably larger than that of the 24-h rhythm, particularly for TF-HRV, seen in both amplitude and phase. The 12-h amplitude of TF-HRV increased up to 574% and 473% during ISS01 and ISS02, respectively, on the first spaceflight, although similar changes were not clear on the second spaceflight.

**Table 3:** Changes of circadian and circasemidian amplitude of heart rate and heart rate variability along with long-duration spaceflight missions.

		Before launch		ISS01		ISS02				Before launch		ISS01		ISS02	
		Mission 1	Mission 2	Mission 1	Mission 2	Mission 1	Mission 2			Mission 1	Mission 2	Mission 1	Mission 2	Mission 1	Mission 2
HR (b/min)	MESOR	72.86	66.61	71.65	68.18	79.42	70.03	β (0.0001–0.01 Hz)	MESOR	− 1.173	1.072	− 1.147	0.942	− 1.070	0.867
	Circadian amplitude	4.40	9.71	**11.22 (255%)**	8.38	**11.93 (271%)**	12.11 (125%)		Circadian amplitude	0.172	0.287	**0.521 (303%)**	0.356	**0.401 (233%)**	0.253
	Circasemidian amplitude	13.2	5.3	6.8	5.5	8.2	7.4		Circasemidian amplitude	0.194	0.127	0.094	0.144	0.329	0.294
NN-interval (ms)	MESOR	853.15	930.80	866.30	897.60	778.60	882.90	LF-component (ms^2^) (0.05–0.15 Hz)	MESOR	1205.8	1097.6	791.6	740.4	655.9	801.0
	Circadian amplitude	48.14	129.80	**128.70 (267%)**	114.20	**119.40 (248%)**	148.50 (114%)		Circadian amplitude	441.8	375.6	20.1	33.7	102.9	254.2
	Circasemidian amplitude	138.3	82.1	83.3	75.9	81.2	89.5		Circasemidian amplitude	352.7	295.0	104.6	36.2	61.1	139.4
r-MSSD (ms)	MESOR	43.45	58.94	42.45	34.68	27.16	43.92	HF-component (ms^2^) (0.15–0.50 Hz)	MESOR	443.5	728.8	319.9	284.5	168.0	485.1
	Circadian amplitude	4.21	29.70	4.17	7.90	1.14	14.37		Circadian amplitude	56.0	715.1	37.6	115.4	18.1	402.5
	Circasemidian amplitude	17.32	24.01	1.41	4.24	0.73	11.60		Circasemidian amplitude	287.8	607.0	26.1	63.4	12.6	307.4
pNN50 ( % )	MESOR	16.86	21.19	18.34	9.52	6.25	12.92	LF-band (ms^2^) (0.01–0.05 Hz)	MESOR	1612.5	2156.2	1117.0	1087.6	827.5	1331.9
	Circadian amplitude	5.40	8.25	3.36	2.99	0.26	2.50		Circadian amplitude	612.2	785.3	295.2	217.0	230.0	605.4
	Circasemidian amplitude	8.27	7.17	1.01	2.92	0.39	4.05		Circasemidian amplitude	930.1	605.9	107.1	216.1	234.6	640.1
LF/HF (–)	MESOR	4.42	3.31	3.60	4.36	5.61	4.01	MF1-band (ms^2^) (0.05–0.10 Hz)	MESOR	763.2	803.1	549.2	498.4	434.1	550.5
	Circadian amplitude	0.58	1.12	0.39	0.85	0.36	0.52		Circadian amplitude	207.4	228.4	56.1	88.4	123.4	171.5
	Circasemidian amplitude	1.26	0.76	0.16	0.85	0.86	1.23		Circasemidian amplitude	217.2	232.0	94.8	39.1	54.9	167.9
TF (ms^2^) (0.0001–0.50 Hz)	MESOR	8012.5	9851.4	8563.9	5578.0	4717.9	6756.6	MF2-band (ms^2^) (0.10–0.15 Hz)	MESOR	442.6	437.9	242.4	245.1	221.8	290.9
	Circadian amplitude	3927.2	3408.7	6385.6 (163%)	2827.0	2152.4	2721.3		Circadian amplitude	234.9	88.9	49.9	66.1	76.1	73.7
	Circasemidian amplitude	311.3	3434.1	**1785.5 (574%)**	1710.7	**1473.0 (473%)**	1167.1		Circasemidian amplitude	140.2	110.4	29.5	42.5	52.8	42.7
ULF (ms^2^) (0.0001–0.003 Hz)	MESOR	4059.4	5071.0	5874.3	2924.9	2717.5	3449.9	HF-band (ms^2^) (0.15–0.20 Hz)	MESOR	181.9	444.2	107.6	102.0	63.8	154.5
	Circadian amplitude	2721.4	3021.9	**6757.7 (248%)**	3060.2	2255.6	3422.8		Circadian amplitude	33.4	529.3	6.1	16.3	9.7	90.6
	Circasemidian amplitude	1712.6	1776.2	1692.6	1683.5	1449.8	2469.7		Circasemidian Amplitude	123.2	460.1	1.9	5.5	5.8	101.8
VLF (ms^2^) (0.003–0.04 Hz)	MESOR	2606.3	3455.9	1582.1	1538.2	1140.9	1856.1	HF02-band (ms^2^) (0.20–0.30 Hz)	MESOR	157.4	300.2	122.6	116.4	63.6	187.0
	Circadian amplitude	1112.3	954.2	304.4	87.7	351.5	756.2		Circadian amplitude	16.8	271.7	**37.1 (221%)**	69.1	17.8	169.8
	Circasemidian amplitude	1520.8	1207.1	114.3	327.7	335.2	929.7		Circasemidian amplitude	99.0	271.7	19.4	40.4	5.7	117.8
LF (ms^2^) (0.04–0.15 Hz)	MESOR	1395.8	1487.1	937.0	874.7	772.1	982.7	HF03-band (ms^2^) (0.30–0.40 Hz)	MESOR	69.6	140.6	55.6	43.6	25.4	92.8
	Circadian amplitude	483.1	382.0	25.2	52.9	122.1	162.1		Circadian amplitude	10.5	142.6	2.6	21.9	1.8	68.4
	Circasemidian amplitude	392.2	390.8	125.5	15.1	84.7	220.3		Circasemidian amplitude	43.5	112.3	6.7	12.5	3.8	54.4
HF (ms^2^) (0.15–0.40 Hz)	MESOR	408.89	909.60	285.70	262.40	152.75	443.30	HF04-band (ms^2^) (0.40–0.50 Hz)	MESOR	34.6	60.7	34.2	23.0	15.2	47.3
	Circadian amplitude	56.82	988.70	40.46	104.60	19.29	286.70		Circadian amplitude	1.0	60.8	**3.1 (317%)**	9.8	1.6	30.1
	Circasemidian amplitude	265.3	821.4	23.9	55.6	12.3	260.5		Circasemidian amplitude	24.2	46.6	5.7	6.4	1.8	19.9

Mission 2 took place about 4 years after Mission 1; ISS01 and ISS02 took place on days 18/21 and 67/181 after launch during mission 1/2, respectively.

Results highlighted in bold indicate changes larger than 200%.

An apparent phase shift of the 24-h and 12-h components of HRV endpoints in response to spaceflight was observed after fitting a single 24-h or 12-h cosine curve separately to the 20 HRV measures by cosinor. Results are summarized in Table 4, where misaligned circadian phases occurring at unusual times (such as day-night reversals), are shown in bold. On the first but not on the second spaceflight, quite a few HRV endpoints show circadian misalignment pre-flight (Table 4, left), suggesting that circadian desynchrony due to social jetlag was larger on the first than on the second mission.

**Table 4:** Circadian and circasemidian phase changes in heart rate variability indices in response to space flight differ between the two long-term missions.

Mission 1				Mission 2			
Circadians	Before	ISS01	ISS02	Circadians	Before	ISS01	ISS02
HR (b/min)	16:55	14:18	15:10	HR (b/min)	15:26	15:01	12:18
NN-interval (ms)	6:22	2:45	3:15	NN-interval (ms)	3:08	2:52	0:46
r-MSSD (ms)	**14:08**	2:59	2:48	r-MSSD (ms)	2:13	1:46	1:32
pNN50 ( % )	**15:34**	2:07	17:56	pNN50 ( % )	3:29	0:28	1:10
LF/HF ratio (–)	16:17	16:35	05:57	LF/HF ratio (–)	13:49	13:13	16:58
LF-component (ms^2^) (0.05–0.15 Hz)	**16:32**	21:10	7:35	LF-component (ms^2^) (0.05–0.15 Hz)	6:27	23:04	2:30
HF-component (ms^2^) (0.15–0.50 Hz)	**14:41**	4:08	4:08	HF-component (ms^2^) (0.15–0.50 Hz)	3:32	2:03	2:43
TF (ms^2^) (0.0001–0.50 Hz)	13:00	12:23	12:09	TF (ms^2^) (0.0001–0.50 Hz)	**10:21**	17:40	09:17
ULF (ms^2^) (0.0001–0.003 Hz)	13:30	12:37	13:01	ULF (ms^2^) (0.0001–0.003 Hz)	**11:11**	17:25	10:36
VLF (ms^2^) (0.003–0.04 Hz)	**14:03**	05:59	07:08	VLF (ms^2^) (0.003–0.04 Hz)	06:13	03:47	01:58
LF (ms^2^) (0.04–0.15 Hz)	**16:15**	00:36	07:06	LF (ms^2^) (0.04–0.15 Hz)	04:09	23:47	00:49
HF (ms^2^) (0.15–0.40 Hz)	**14:39**	04:02	04:21	HF (ms^2^) (0.15–0.40 Hz)	01:56	01:52	01:54
LF-band (ms^2^) (0.01–0.05 Hz)	**13:52**	04:25	05:27	LF-band (ms^2^) (0.01–0.05 Hz)	04:45	03:54	02:08
MF1-band (ms^2^) (0.05–0.10 Hz)	**16:44**	01:17	05:03	MF1-band (ms^2^) (0.05–0.10 Hz)	03:55	00:58	01:07
MF2-band (ms^2^) (0.10–0.15 Hz)	16:21	14:40	13:18	MF2-band (ms^2^) (0.10–0.15 Hz)	**05:20**	14:24	14:17
HF-band (ms^2^) (0.15–0.20 Hz)	**15:51**	09:19	09:07	HF-band (ms^2^) (0.15–0.20 Hz)	01:53	03:40	02:37
HF02-band (ms^2^) (0.20–0.30 Hz)	**12:53**	03:27	02:43	HF02-band (ms^2^) (0.20–0.30 Hz)	02:30	01:44	02:20
HF03-band (ms^2^) (0.30–0.40 Hz)	**13:37**	03:33	22:29	HF03-band (ms^2^) (0.30–0.40 Hz)	01:43	01:58	02:20
HF04-band (ms^2^) (0.40–0.50 Hz)	00:37	14:51	18:55	HF04-band (ms^2^) (0.40–0.50 Hz)	01:38	01:11	01:40
β	06:32	01:36	02:10	β	01:36	04:38	23:11

Mission 1				Mission 2			
Circasemidians	Before	ISS01	ISS02	Circasemidians	Before	ISS01	ISS02
HR (b/min)	− 355	− 328	− 312	HR (b/min)	− 292	− 306	− 305
NN-interval (ms)	− 82	− 238	− 225	NN-interval (ms)	− 207	− 37	− 211
r-MSSD (ms)	− 93	− 20	− 235	r-MSSD (ms)	− 207	− 16	− 236
pNN50 ( % )	− 98	− 359	− 316	pNN50 ( % )	− 212	− 357	− 247
LF/HF ratio (–)	− 9	− 352	− 6	LF/HF ratio (–)	− 299	− 271	− 342
LF-component (ms^2^) (0.05–0.15 Hz)	− 98	− 9	− 10	LF-component (ms^2^) (0.05–0.15 Hz)	− 215	− 356	− 229
HF-component (ms^2^) (0.15–0.50 Hz)	− 93	− 205	− 284	HF-component (ms^2^) (0.15–0.50 Hz)	− 223	− 17	− 242
TF (ms^2^) (0.0001–0.50 Hz)	− 320	− 320	− 302	TF (ms^2^) (0.0001–0.50 Hz)	− 246	− 92	− 302
ULF (ms^2^) (0.0001–0.003 Hz)	− 4	− 323	− 310	ULF (ms^2^) (0.0001–0.003 Hz)	− 255	− 97	− 316
VLF (ms^2^) (0.003–0.04 Hz)	− 93	− 288	− 258	VLF (ms^2^) (0.003–0.04 Hz)	− 240	− 62	− 241
LF (ms^2^) (0.04–0.15 Hz)	− 281	− 11	− 194	LF (ms^2^) (0.04–0.15 Hz)	− 209	− 19	− 227
HF (ms^2^) (0.15–0.40 Hz)	− 92	− 211	− 280	HF (ms^2^) (0.15–0.40 Hz)	− 206	− 16	− 233
LF-band (ms^2^) (0.01–0.05 Hz)	− 92	− 240	− 243	LF-band (ms^2^) (0.01–0.05 Hz)	− 230	− 62	− 236
MF1-band (ms^2^) (0.05–0.10 Hz)	− 94	− 17	− 217	MF1-band (ms^2^) (0.05–0.10 Hz)	− 208	− 29	− 221
MF2-band (ms^2^) (0.10–0.15 Hz)	− 284	− 337	− 342	MF2-band (ms^2^) (0.10–0.15 Hz)	− 214	− 320	− 260
HF-band (ms^2^) (0.15–0.20 Hz)	− 92	− 8	− 281	HF-band (ms^2^) (0.15–0.20 Hz)	− 206	− 37	− 235
HF02-band (ms^2^) (0.20–0.30 Hz)	− 91	− 221	− 262	HF02-band (ms^2^) (0.20–0.30 Hz)	− 38	− 15	− 238
HF03-band (ms^2^) (0.30–0.40 Hz)	− 96	− 6	− 309	HF03-band (ms^2^) (0.30–0.40 Hz)	− 207	− 19	− 250
HF04-band (ms^2^) (0.40–0.50 Hz)	− 284	− 354	− 322	HF04-band (ms^2^) (0.40–0.50 Hz)	− 204	− 5	− 264
β	− 90	− 32	− 230	β	− 206	− 27	− 45

Bold cells mean circadian phase misalignment induced by astronaut social jetlag on Earth before spaceflight mission.

Circasemidian acrophases expressed in (negative) degrees, with 360° ≡ 12 h, 0° = 00:00.

**Figure 3:**
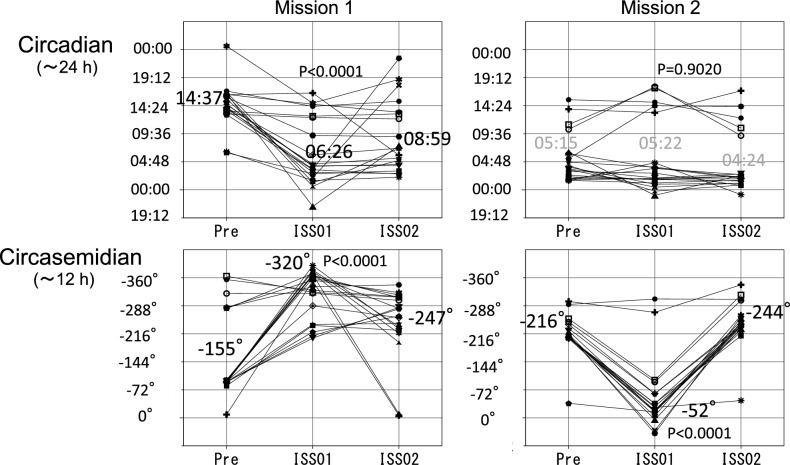
Changes in 24-h and 12-h acrophases of HRV endpoints during two space missions. On the first mission (Mission 1, left), circadian acrophases advanced on average by about 8 h from pre-flight to ISS01 (from 14:37 to 06:26, P < 0.0001). Circadian acrophases were mostly restored during ISS01 and ISS02, when they averaged 08:59 (top left). Circasemidian acrophases were delayed on average by 165° (5.5 h) from − 155° (05:10 and 17:10) to − 320° (10:40 and 22:40) (P < 0.0001) between pre-flight and ISS01 (bottom left). On the second spaceflight (Mission 2, right), circadian acrophases showed no significant changes on average from Pre (05:15) to ISS01 (05:22) and ISS02 (04:24). Circasemidian acrophases were phase-advanced on average by164° (about 5.5 h) from − 216° (07:12 and 19:12) to − 52° (01:44 and 13:44) (P < 0.0001), returning to their original acrophase of − 244° (08:08 and 20:08) during ISS02 (bottom right). Acrophases of 12-h component expressed in (negative) degrees, with 360° ≡ 12 h, 0° = 00:00.

The recovery process of such internal desynchrony of the 20 HRV indices during spaceflight is illustrated in Fig. 3. It depicts the time course of the circadian (Fig. 3, top) and circasemidian (Fig. 3, bottom) acrophases during the first (Fig. 3, left) and second (Fig. 3, right) missions. On the first mission, circadian acrophases advanced on average by about 8 h, from 14:37 (pre-flight) to 6:26 (ISS01). Any misalignment of these circadian acrophases improved during ISS01, maintaining a similar timing of 8:59 during ISS02 (Fig. 3, top left). Circasemidian acrophases showed an average phase-delay of 165° (5.5 h) from − 155° (05:10 and 17:10) to − 320° (10:40 and 22:40) during ISS01, and were maintained at − 247° (08:14 and 20:14) during ISS02 (Fig. 3, bottom left). The 5-time amplified 12-h component may thus help restore internal synchrony to the 24-h clock. Presumably, circadian acrophases may have over-adjusted to 06:26 during ISS01 and were readjusted to 08:59 during ISS02 (Fig. 3, top left).

During the second mission, circadian acrophases, on average, showed no significant changes from pre-flight (05:15) to ISS01 (05:22) and ISS02 (04:24) (Fig. 3, top right). Circasemidian acrophases, by contrast, advanced on average by 164° (5.5 h) from − 216° (07:12 and 19:12) to − 52° (01:44 and 13:44) (P < 0.0001), returning to their original phase of − 244° (08:08 and 20:08) during ISS02 (Fig. 3, bottom right). As observed previously^20^, the 12-h component may help restore appropriate circadian timing in the presence of desynchrony. Mapping the circadian and circasemidian amplitude and phase characteristics could thus serve as important markers of adaptation to the space environment.

## Discussion

Repeated HRV monitoring over 24 h of a healthy astronaut during two space missions, 4 years apart, served to illustrate methods to assess changes in brain plasticity and psychological resilience. Whereas conclusions cannot be derived from data from a single astronaut monitored for only 24 h at discrete times before, during and after a mission in space, the methodology used herein could be used in future studies to determine whether neural adaptation improves on repeated missions, as observed herein around day 20 after launch. During nighttime, sleep improved, and HRV activity co-varying with brain neural activity in the SN accelerated, while decelerating during daytime. HRV endpoints reflecting DMN activity showed no differences between the two space missions.

### Stimulating environment and brain plasticity

Brain plasticity refers to the capacity of neurons and of neural circuits in the brain to change, structurally and functionally, in response to experience. This property is fundamental for the adaptability of behavior, for learning and memory processes, brain development, and brain repair. Exposure to stimulating environments has repeatedly been shown to strongly influence brain plasticity. Thus, it is a crucial underlying component of the enormous challenge of space adaptation for astronauts. Neural plasticity can take place at several levels, from synaptic plasticity at the (sub)cellular level to plasticity at the system and network levels^44–46^. Brain plasticity can be studied with a number of methods, such as electroencephalography (EEG)/evoked potentials (ERPs), structural and functional MRI and transcranial magnetic stimulation (TMS). In addition to functional brain response^47–50^ illustrated herein, recent work showed structural changes in the brain after long-duration space flights^4–7^ resulting from alterations in sleep performance or functional brain networks. Both aspects were estimated by HRV in a healthy astronaut who also took part in repeated space missions^20,21,28^.

Our observation of improved sleep agrees with results from a previous study^28^ where we assessed sleep quality based on sleep-related changes in RR-intervals and HRV-HF, which we found to be improved in space, and to be associated with increased parasympathetic activity, contrary to previous investigations^51–54^. Sleep quality was assessed as changes in sleep performance and HRV behavior in specific frequency regions for interpretation in terms of functional brain networks, as done in previous studies^20,25,26,28,42^. Previous investigations reported shorter sleep duration and inadequate sleep quality of astronauts during spaceflight aboard the ISS. These results were attributed to environmental factors, including exposure to microgravity, the 90-min light–dark cycle from the skylight weightlessness itself, excitement, and workload scheduled by operational demands^53,54^.

### Effect of nighttime HRV changes on brain plasticity in space

Despite increased interest in the effect of spaceflight on the human central nervous system (CNS)^15,55^, not much is known thus far about the functional and morphological effects of microgravity on the human CNS. Previous studies have shown that CNS changes occur during and after spaceflight in the form of neuro-vestibular problems, alterations in cognitive function and sensory perception, problems with motor function, cephalic fluid shift, and psychological disturbances^56,57^. In the past few years, advances in structural and functional neuroimaging techniques have shown spaceflight-induced neuroplasticity in humans in several brain regions, including the insular cortex, the temporo-parietal junction, and the thalamus, in relation to short- and long-duration spaceflight^1,2,14^.

HRV indices that co-vary with SN activity are of particular interest since the SN is linked to the autonomic nervous system function and is sensitive to environmental challenges. The SN is mainly centered on the dorsal anterior cingulate, extending into the perigenual anterior cingulate cortex, and orbital fronto-insular cortices, but it also encompasses the limbic and brainstem areas. Relevance to HF-HRV is suggested by the inclusion of known autonomic nervous system control areas in the SN, and by this vagal marker’s putative role in switching between rest and activity and between internal and external focus of attention.

Further investigations are needed to examine whether acceleration of SN activity starts with nighttime sleep, as observed herein. It would suggest that brain plasticity may be initiated at night. The sensitivity of vagally-induced heart rate reactions to event salience might further suggest relationships between the SN and HF-HRV, as might the apparent overlap between nodes of the SN and areas related to autonomic control.

Identified as related to HF-HRV, the mPFC is important both as a node in the DMN and in the SN^58^. Anatomically, the mPFC is known to connect to pre-autonomic cell groups in the hypothalamus, periaqueductal gray, and brainstem^59,60^. If diffuse attention is a major aspect of the functionality of the DMN, then the overlapping membership of the mPFC in the two networks would provide an anatomical site for shifting from DMN activation to SN activation. Some evidence supports the view that DMN activation is switched to SN activation when an interoceptive or environmental stimulus is encoded as significant^61^.

### Daytime HRV fluctuations associated with brain resilience in space

Because HRV may be associated with neural structures that are involved in the appraisal of threat and safety, HRV can be considered a potential marker of stress. HRV reflects the status of one’s ongoing adjustment to constantly changing environmental demands. Previously, under stressful environments, such as performing tasks during a spaceflight mission, HRV was found to be decreased^17^. Increased HF-HRV is considered to be associated with a positive mood, absence of negative affect, and an alert readiness to engage with the physical and social environment^62,63^.

Much recent research has found that psychological resilience is mediated by spontaneous brain activity measured with resting-state functional MRI. Although Waugh et al.^64^ found that when faced with a threat, participants had prolonged changed activity in the insula in response to aversive stimuli, psychological resilience is a complex construct that likely involves different brain functions. Other studies provided evidence that brain resilience is related not only to the insula, but also to the mPFC, OFC, PCC, ACC, and thalamus^65–71^. In the extant literature, the most consistent brain area related to psychological resilience is the ACC, perhaps because the ACC is associated with many important emotional functions, including motivation, emotion regulation, and attention or adaptation to a novel environment, such as space^72–75^. Previous investigations on resilience speculated that local activity in the ACC (such as fractional amplitude of low-frequency fluctuations measured by fMRI) would be negatively associated with psychological resilience^23,75,76^.

The bi-directional connections between heart and brain enunciated by Claude Bernard can be studied by analyzing HRV^17,77^. Over the past several years, many neuroimaging studies examined the association of HRV endpoints with fluctuations in brain functional connectivity^18,19,32–35,59,60,78^. They confirmed the existence of intimate connections between the different brain regions and HRV endpoints. They also posited that any changes in brain functional networks, which dynamically adjust the structure of their global and local network connectivity, should affect and change HRV activities in their respective frequency bands. “HRV is like a mirror reflecting the strength of activities of humans’ brain and mind”^17,19,77^. 

Astronauts’ motivation aboard the ISS is also expected to reflect changed activities in the respective HRV frequency bands. Several investigations reported a relation between levels of psychological well-being and HRV^35,79^, which confirmed a statistically significant negative correlation between life satisfaction and HF-HRV activities^35^. Should our observation of decreased spectral power of HF-HRV, HF-component, and the series of the HF-band groups, and of lowered r-MSSD, pNN50 and Lorenz plot’s measures (Table 2, left and Fig. 2, left) be confirmed in future studies, it would suggest psychological resilience on repeated space missions.

### Role of biological rhythms in the adaptation to the space environment

Whereas the circadian system plays a key role in the adaptation to a novel environment, such as microgravity in space^19,20,25–28^, ultradian components provided an evolutionary advantage for almost all life forms, from bacteria to humans^80–84^.

These ultradian rhythms can be expected to be important for the rapid adaptation to microgravity in space. The 12-h (circasemidian) component in particular may be involved^85–90^. It may reflect the function of two stress response pathways reacting to unfolded protein in the endogenous endoplasmic reticulum (ER) and mitochondria. A 12-h (circasemidian) component characterizes the ER- and mitochondria-associated “unfolded protein response (UPR) cycle”^88–93^. Several potential roles of the circasemidian clock in coordinating human health have been proposed, such as maintaining metabolic homeostasis^87^, coordinating sleep quality of slow wave sleep^94,95^, and mediating aging, especially in the prevention of aging-related metabolic decline^87,88,96,97^.

Based on our observations herein, the following hypothesis comes to mind. First, when faced with a new environment in space, the 12-h response appears faster and is larger than the circadian response (Table 3). Second, strong 12-h clock regulation might help repair circadian desynchrony (Table 4 and Fig. 3). The more severe internal desynchrony is (Table 4, Flight 1), the larger is the activation of the 12-h component (Table 3 and Fig. 3, Flight 1). Third, a milder circasemidian response during the second than during the first mission suggests that spaceflight-induced neuroplasticity may be present in the astronaut’s brain during the second mission.

Harmonic oscillations of 24 and 12 h likely provide evolutionarily adaptive advantages. The 12-h (circasemidian) component may contribute to consolidating a strong circadian system in space, and may contribute to a better adaptation in space by taking advantage of brain plasticity at night and psychological resilience during daytime.

### Limitations

This investigation has several limitations. First, the study is limited to a single astronaut, and results were only compared between missions on a single day (ISS01). Factors other than adaptation to space environment (such as exercise, nutrition, mission tasks, and interpersonal stress) likely contributed in part to the results. As such, results herein do not provide inferential information about the effect of repeated missions of many days flown by a “population” of astronauts. Future studies should be designed to also estimate the uncertainty due to variation between astronauts and between mission days for each astronaut.

Despite the medium to large effect size of changes observed in this illustrative case, serial correlation, reduced by considering hourly averages instead of the original 5-min HRV endpoints, remains an issue preventing the derivation of generalizable inferences. As similar data from other astronauts become available, individual estimates can be used as imputations that no longer depend on the sampling interval.

In view of the importance of the circadian rhythm, there is merit in recording ECG around the clock. Demanding schedules and inconvenience of implementing the monitoring have been limiting factors to obtaining more data or data covering spans longer than 24 or 48 h. As technology advances, ECG monitors may become easier to use for longer spans, and as space exploration expands, more space travelers may participate in similar studies in the future.

Space adaptation of human neural cardiovascular coordination remains a challenge, as mechanisms are diverse and complex. Second, brain oscillatory activity data are lacking. Several studies, however, showed that HRV is associated with structures and functions of the neural network, and HRV is a biomarker reflecting activities of the brain integration system. These associations are extremely complex, however, and have not yet been fully confirmed. Future investigations are needed to directly assess the brain’s oscillatory activity in space. The methodology used in this investigation may help address these complex issues in future studies.

## Conclusion

We examined the hypothesis proposed by Demertzi et al.^14^ that second-time flyers adapt more quickly and are less prone to microgravity-induced problems^14,16,21^. This demonstration is a simple illustration of methodology aimed to assess changes in brain plasticity and psychological resilience in a single astronaut, limited to comparing HRV endpoints between missions on a single day. Results nevertheless confirm earlier findings that sleep duration lengthened and sleep quality improved in space. The methodology used herein outlines how HRV behavior, which estimates the process of neural adaptation^17,20,21,28^, can serve to interpret changes in terms of brain functional networks. In the case examined herein, we find that brain plasticity during nighttime and psychological resilience during daytime may help with the adaptation to space’s environment. The 12-h component may have played a role in the adaptation process since it underwent larger changes than the 24-h component in response to the space environment, as assessed around day 20 on the ISS. HRV in the HF spectral region may be critical to assess microgravity-induced brain plasticity and psychological resilience, because HF-HRV reflects the adaptation process. Further studies are needed to examine how adaptation to the microgravity environment in space occurs. The role of functionally integrating the SN, consisting of neural centers (ACC, OFC, Amygdala and Insula), which involves and responds in a task-dependent manner to interceptive-autonomic and reward processes in a task-independent manner to emotional and homeostatic stimuli of personal salience^23,35,71,72,75,98–100^ may be particularly important, as our data suggest.

## Data Availability

Restrictions from Japan’s Aerospace Exploration Agency (JAXA) apply to the availability of the data supporting the findings of this study. The data were used under license for the current study. Although data are not publicly available, they are available to collaborating parties under ethical approval from JAXA.
